# Effect of Topical Calcium Channel Blockers on Intraocular Pressure in Steroid-induced Glaucoma

**DOI:** 10.5005/jp-journals-10008-1155

**Published:** 2014-01-16

**Authors:** Sunil Ganekal, Syril Dorairaj, Vishal Jhanji, Krishnaprasad Kudlu

**Affiliations:** Assistant Professor, Department of Ophthalmology, JJM Medical College Davangere, Karnataka, India; Associate Professor, Department of Ophthalmology, Mayo Clinic, Jacksonville Florida, USA; Assistant Professor, Department of Ophthalmology and Visual Sciences, Chinese University of Hong Kong, Hong Kong; Medical Director, Department of Ophthalmology, Prasad Nethralaya, Udupi Karnataka, India

**Keywords:** Calcium channel blockers, Intraocular pressure, Steroid-induced glaucoma.

## Abstract

**Purpose:** To evaluate the effect of 0.125% verapamil and 0.5% diltiazem eye drops on intraocular pressure (IOP) in steroid-induced glaucoma in rabbit eyes.

**Methods:** A total of 18 rabbits with steroid-induced glaucoma were divided into three groups (A, B and C; n = 6 each). Right eyes in groups A, B and C received 0.5% diltiazem, 0.125% verapamil and 0.5% timolol eye drops twice daily for 12 days, respectively; whereas, left eyes received distilled water. IOP was measured with Tono-pen XL at baseline, day 4, day 8, and day 12 of treatment.

**Results:** Both 0.5% diltiazem and 0.125% verapamil eye drops significantly reduced IOP compared to control eyes (p < 0.05). Reduction of IOP by 0.5% diltiazem, 0.125% verapamil eye drops were comparable to 0.5% timolol. No surface toxicity or systemic side effects were noted during the study period.

**Conclusion:** Calcium channel blockers, verapamil, and diltia-zem significantly reduced IOP in rabbiteyes. This group of drugs may have a potential role in treatment of glaucoma

**How to cite this article:** Ganekal S, Dorairaj S, Jhanji V, Kudlu K. Effect of Topical Calcium Channel Blockers on Intraocular Pressure in Steroid-induced Glaucoma. J Current Glau Prac 2014;8(1):15-19.

## INTRODUCTION

Glaucoma is second leading cause of blindness worldwide.^1^ Characterized by progressive degeneration of retinal ganglion cells and optic nerve fibers, leading to gradual deterioration of visual field. If untreated, it can lead to irreversible blindness.^2^ In most of the cases, glaucoma is associated with high intraocular pressure (IOP). Prophylactic medical reduction of IOP reduces the risk of progression to glaucoma from ~10 to 5%.^3^ There is a constant search for newer drugs that can lower the IOP and therefore possibly retard the progression of glaucomatous optic nerve damage.

Calcium is an important intracellular messenger and Ca^2+^ infux could have several effects on aqueous humor dynamics, including hydrostatic component, ciliary perfusion and osmotic component.^4^ Calcium channel blockers (CCBs), which are commonly used for the treatment of hypertension and coronary vascular disease, reduce the tone of blood vessels by inhibiting Ca^2+^ infux, causing vasodilation and incre a sing regional blood fow in several organs including the optic nerve head.^5-10^

Calcium channel blockers may also inhibit the synthesis of extracellular matrix collagen protein, suggesting beneficial effect in glaucoma.^13^ CCBs cause relaxation of trabecular mesh work cells by inhibition of L-type channels which increases outfow facility of aqueous humor. The perfusion studies in dissected human eyes showed dose-related increase in outfow facility after verapamil administration.^11,12^

In the present study, we investigated the ocular hypo-tensive role of CCBs in rabbit eyes.

## METHODS

The holding and experimental protocols were conducted in accordance with the Association for Research in Vision and Ophthalmology Statement for the Use of Animals in Ophthalmic and Vision Research. The study protocol was approved by the ethics committee of JJM Medical College, Karnataka. A total of 18 albino rabbits (aged 3-4 months) of either sex weighing 1.5 to 2.5 kg were used in this study. The rabbits were inbred in the central animal house under suitable conditions of housing, temperature, ventilation and nutrition. All IOP measurements were obtained with Tono-pen XL (Reichert Technologies) after anesthetizing the rabbits with 5 mg/ml intravenous midazolam given in dose of 0.5 to 1 mg/kg through marginal ear vein. In addition, topical anes thesia in the form of lignocaine hydrochloride was used before each IOP measurements. An average of three IOP readings was used. Ocular hypertension was induced by bilateral instillation 1% predisolone acetate eye drops twice a day for a period of 40 days. IOP measurements were obtained before and after treatment with topical corticoste-roid eye drops. Subsequently, the rabbits were divided into three groups and all right eyes in each group received twice daily diltiazem 0.5% eye drops (group A; n = 6) or verapamil 0.125% eye drops (group B; n = 6) or timolol maleate 0.5% eye drops (group C; n = 6) twice daily for 12 days. Sterile distilled water was used twice daily in all left eyes. Diltiazem 0.5% eye drops were prepared by diluting injection diltiazem 25 mg/ml with distilled water upto a concentration of 5 mg/ml. Verapamil 0.125% eye drops were prepared by diluting injection verapamil 2.5 mg/ml with distilled water to a concentration of 1.25 mg/ml.

IOP was measured in both eyes before instilling these drugs and on every 4th day till the end of 12 days of treatment period.

### Statistical Analysis

Results were expressed as mean ± SD and percentage changes wherever required. Intragroup comparisons were performed using the t-test. One-way analysis of variance was used for multiple group comparisons followed by post hoc Tukey's test for group-wise comparisons. A ‘p' value of 0.05 or less was considered for statistical significance.

**Fig. 1:**
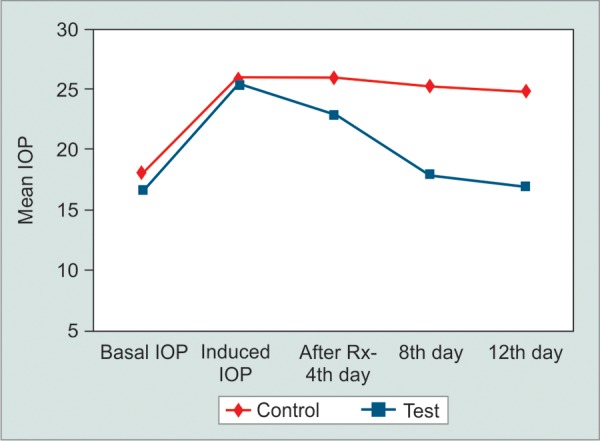
Intraocular pressure changes in treatment and control eyes during study period in 0.5% diltiazem treated rabbits (group A)

**Fig. 2:**
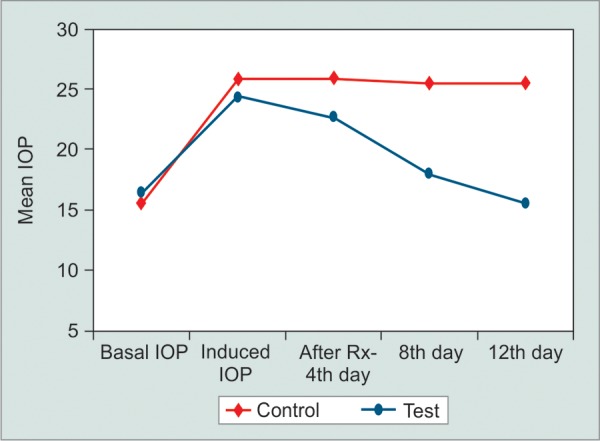
Intraocular pressure changes in treatment and control eyes during study period in 0.125% verapamil treated rabbits (group B)

**Fig. 3:**
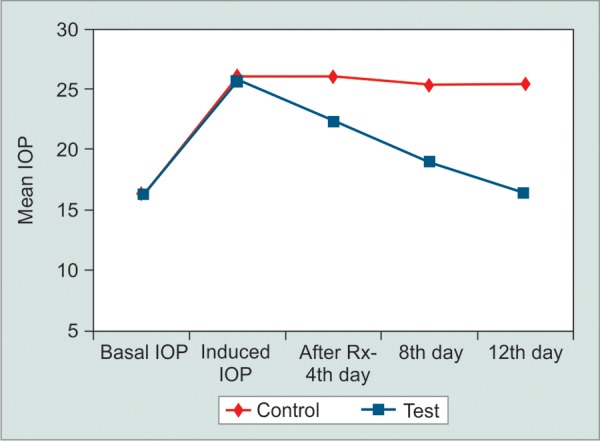
Intraocular pressure changes in treatment and control eyes during study period in 0.5% timolol treated rabbits

**Fig. 4:**
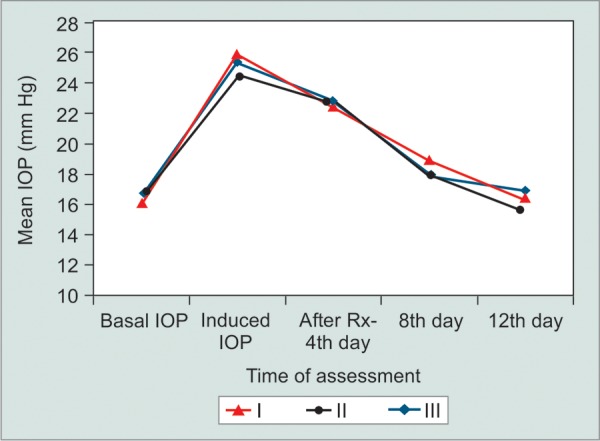
Comparison of intraocular pressure changes in right (treatment) eyes in I-0.5% diltiazem, II-0.125% verapamil and III-0.5% timolol-treated rabbits during study period

## RESULTS

Mean basal IOP increased in all three groups after 40 days of twice daily treatment with 1% prednisolone acetate eye drops (Tables 1 to 3). All groups were comparable in terms of pre- and postcorticosteroid treatment (p > 0.05). Group A (diltiazem 0.5%) eyes did not show any statistically signif-cant reduction in the IOP in the left eyes (controls) up to day 12. However, the IOP reduced in the right eyes (treatment) starting from day 4 as shown in Figure 1. There was a statistically significant difference in the mean IOP treatment and control eyes in group A (p = 0.0153). The control eyes in group B did not show a significant reduction in the IOP over the study period. The treatment eyes showed a signif-cant reduction in the mean IOP level on days 4, 8 and 12 (p = 0.0171) (Fig. 2). Similar results were obtained in the treatment and control eyes of group C (p = 0.0192) (Fig. 3). Further, there was no statically significant difference in the IOP lowering effect of all three drugs (post hoc Tukey's test) (Fig. 4). During the study period, no ocular surface toxicity or systemic side effects were noted in any of the rabbits.

**Table 1:** **Table 1:** Mean basal, post-topical corticosteroid and post-topical diltiazem treatment intraocular pressure in group A rabbits

		*Baseline*		*After corticosteroid*		*Day 4*		*Day 8*		*Day 12*	
OD diltiazem		16.4 ± 1.4		25.5 ± 1.6		22.9 ± 1.7		17.9 ± 1.3		16.9 ± 1.1	
OS control		18.0 ± 2.3		25.9 ± 1.9		25.9 ± 1.9		25.2 ± 1.7		24.8 ± 1.7	

**Table 2:** **Table 2:** Mean basal, post-topical corticosteroid and post-topical verapamil treatment intraocular pressure in group B rabbits

		*Baseline*		*After corticosteroid*		*Day 4*		*Day 8*		*Day 12*	
OD verapamil		16.4 ± 1.4		24.5 ± 1.0		22.7 ± 1.3		18.0 ± 2.3		15.5 ± 1.4	
OS control		15.5 ± 1.4		25.9 ± 1.9		25.9 ± 1.9		25.5 ± 1.6		25.5 ± 1.6	

**Table 3:** **Table 3:** Mean basal, post-topical corticosteroid and post-topical timolol treatment intraocular pressure in group C rabbits

		*Baseline*		*After corticosteroid*		*Day 4*		*Day 8*		*Day 12*	
OD timolol		16.0 ± 1.5		22.4 ± 1.9		22.4 ± 1.9		19.0 ± 1.8		16.4 ± 1.4	
OS control		16.4 ± 1.4		26.2 ± 2.1		26.2 ± 2.1		25.5 ± 1.6		25.5 ± 1.6	

## DISCUSSION

Most of the previous studies have employed normal/low tension glaucoma animal models to demonstrate the effects of topical CCBs on IOP. In the present study, we demonstrated a reduction in corticosteroid-induced ocular hypertension with topical calcium channel blocking drugs. The hypotensive effect was comparable to that of topical timolol eye drops.^13^

Calcium channel blockers alter the intracellular calcium concen tration by modifying calcium fux across cell membranes and affect various intracellular signaling processes.^14,15^ Lipid soluble CCBs act at the central nervous system level, whereas water soluble CCBs act mainly on the cornea and optic nerve.^16^ It is also known that calcium infux is the terminal step in axonal death in the glutamate path way. The ability to block calcium infux can, therefore, produce a neuroprotective benefit.^17^ Furthermore, CCBs can improve ocular blood fow through inhibition of endothelin-1.^18-21^ Despite this, the effect of CCBs on IOP remains controversial.^22-27^

Calcium infux could have several effects on aqueous humor dynamics, including a hydrostatic component caused by an effect on arterial blood pressure and ciliary body perfu -sion, and an osmotic component caused by an effect on the active secretion of sodium, calcium and other ions by ciliary epithelium.^28^ Recent reports have addressed the effect of CCBs on ocular blood fow. Using laser Doppler velocimetry and fowmetry in cats, Harino et al demonstrated increased optic nerve head blood fow following administration of intravenous nicardipine.^29^ Netland et al utilized color Dop-pler ultrasound analysis and found that topical verapamil may decrease the vascular resistance in ocular blood vessels.^26^

Favorable effects of CCBs on visual field defects as well as contrast sensitivity have also been reported.^29-31^ Verapamil tends to block both activated and inactivated L-type calcium channels. It has also been shown to improve the blood supply in rabbit eyes with experimental glaucoma acting as vasodilator and improving the outfow facility.^32^ Diltiazem, on the contrary, has been shown to produce relaxation of serotonin-induced contraction of bovine ophthalmic artery primarily by inhibiting the Ca^2+^ infux.^33^ It was shown to exhibit a long lasting and dose-related effect on IOP.^34^ CCBs may, therefore, play a potential role in relaxing the retinal, long posterior ciliary, and ophthalmociliary arteries to improve the ocular circulation in vascular diseases in which considerable vascular tone is present.^35^ Santafe et al reported that CCBs decrease aqueous humor secretion in addition to causing a slight but significant reduction in tomographic outfow facility.^34^ Also, the outfow of aqueous humor infuenced by episcleral venous pressure may be directly affected by calcium inhibition. Verapamil may interfere with gap junctions between nonpigmented and pigmented ciliary epithelial cells altering cellular permeability of the ciliary epithelium and thus inhibiting normal aqueous humor formation.^34,36^ It may also alter the cyclic adenosine monophosphate content in ciliary epithelial cells, thereby affecting IOP through a decrease in aqueous humor formation, or an increase in outfow facility.^37^

Lowering of lOP by verapamil and diltiazem may be due to inhibition of the intracellular uptake of calcium by inactivating the inner phosphorylation-dependent calcium gate of the cellular membrane.^10^ It is known that trabecular meshwork cells have contractile properties, which may be infuenced by Ca^2+^ infux through voltage-dependent L-type Ca^2+^ channels. These agents cause relaxation of trabecular meshwork cells and increase the outfow facility. The perfu-sion studies in dissected human eyes showed dose-related increase in outfow facility after verapamil administration.^38^

Calcium channel blockers cause vasodilatation and reduce vascular resis tance, increase the capillary blood speed in the optic nerve head, this make them to be possible drugs useful in the treatment of low-tension glaucoma.^10^ The results of our study match the earlier reports that showed that topical application of verapamil and diltiazem effectively lowered IOP in a dose-related fashion.^24,34^

Topical verapamil has also been shown to reduce IOP in humans.^7,26,39^ A single topical application of 0.125% vera-pamil prompted a 3 to 4 mm Hg IOP decrease in 12 ocular hypertensive patients that lasted up to 10 hours,^7^ whereas a slight reduction (≈1.5 mm Hg) was noted in normal volun-teers.^26^ After topical application of 0.125% verapamil for 2 weeks, a 7.0 ± 2.9 mm Hg decrease in IOP has been measured in ocular hypertensive subjects.^8^

Our study highlights the potential role of CCBs in management of corticosteroid-induced glaucoma in rabbit eyes. CCBs were comparable with commonly used beta blocker drug. Nevertheless, further studies are needed to replicate the ocular effects of CCBs in humans and determine their potential clinical use in glaucoma patients.
